# Identifying and Characterizing the Circular RNAs during the Lifespan of Arabidopsis Leaves

**DOI:** 10.3389/fpls.2017.01278

**Published:** 2017-07-20

**Authors:** Tengfei Liu, Li Zhang, Geng Chen, Tieliu Shi

**Affiliations:** The Center for Bioinformatics and Computational Biology, Shanghai Key Laboratory of Regulatory Biology, The Institute of Biomedical Sciences and School of Life Sciences, East China Normal University Shanghai, China

**Keywords:** circRNAs, leaves, growth, senescence, regulation

## Abstract

Leaf growth and senescence are controlled by tight genetic factors involved regulation at multiple levels. Circular RNAs (circRNAs) have recently been reported as the microRNA sponge to accomplish corresponding regulatory roles. This study aims to explore the expression profile and functional role of circRNAs in Arabidopsis leaf growth and senescence. We used publically available RNA-seq data of Arabidopsis leaves to identify the circular RNA expression profile and used quantitative real-time PCR to validate our identified circRNAs. The functions of circRNAs were explored using distinct bioinformatics methods including analysis of network, gene ontology and KEGG pathway. We identified 168 circRNAs, including 40 novel circRNAs, in *Arabidopsis thaliana* leaves, with 158 (94.1%) circRNAs arising from the exons of genes. Real-time PCRs were used to verify 4 highly expressed circRNAs and they all showed consistent expression patterns with the RNA-seq results. Interestingly, 6 and 35 circRNAs were differentially expressed at G- to -M stage and M- to -S stage, respectively. The circRNAs display an upregulation trend during the lifespan of Arabidopsis leaves. Moreover, the expression of circRNAs during senescence is independent of host gene expression to a certain degree. The gene ontology (GO) and KEGG pathway analysis of the targeted mRNA of circRNA–miRNA–mRNA network showed that the circRNAs may be involved in plant hormone signal transduction, Porphyrin and chlorophyll metabolism during leaves senescence. Our comprehensive analysis of the expression profile of circRNAs and their potential functions during leaf growth and senescence suggest that circRNAs may function as new post-transcriptional regulators in the senescence of Arabidopsis leaves.

## Introduction

The leaf is an important organ of plants. As primary producers in the ecosystem, leaves fix carbon using light energy and produce food for other species (Woo et al., 2016). Leaves undergo a series of developmental, physiological and metabolic shift throughout their lifespans in an orderly manner (Kim et al., 2016). During the growth stage, leaves use photosynthesis to accumulate chemical energy and nutrients. During the senescence stage, leaf cells undergo dramatic changes in metabolism and cellular structures. Senescence is a very complicated genetic process and involves multiple layers of complex regulation.

Circular RNAs (circRNAs) are a major class of non-coding RNAs that exist ubiquitously in the eukaryotic tree of life. CircRNAs can arise from exons, introns and intergenic regions. Generally, circRNAs are mainly generated by back-splicing events from exons of protein-coding genes where a downstream 3′ splice site joins with an upstream 5′ splice site (Gruner et al., 2016). Moreover, some circRNAs are conserved and exhibit cell-type, tissue-specific or developmental-stage expression (Memczak et al., 2013; Salzman et al., 2013; Gao et al., 2015), suggesting the potential regulatory roles of circRNAs. Some previous studies revealed that circRNAs can regulate gene expression at the transcriptional and posttranscriptional levels (Ashwal-Fluss et al., 2014; Li et al., 2015; Wang, 2015). Furthermore, circRNAs could serve as miRNA sponges by sequestering and preventing microRNAs from binding corresponding target genes (Hansen et al., 2013; Liu Q. et al., 2016; Peng et al., 2016). Nevertheless, the biogenesis and function of circRNAs remains to be studied (Chen and Shan, 2015).

The exploration of circRNAs in plants was just began (Meng et al., 2016). The first discovery of circRNAs in plants was in Wang et al. (2014) and substantial circRNAs were detected in Arabidopsis transcriptome. Plant circRNAs have conservation feature, whereas distinct features of circRNAs were found between plants and animals (Ye et al., 2015). Additionally, two studies revealed that circRNAs can act as a negative regulator for their parental genes, which provides new biological insights into the rice circRNAs (Lu et al., 2015; Darbani et al., 2016). A recent study reported a possible connection between the regulations of circRNAs as miRNA sponge with the expressions of functional genes in wheat leaves associated with dehydration resistance (Wang et al., 2016).

To explore the quantity of circRNAs in lifespan of Arabidopsis leaves and their potential function in the regulation of development and senescence of leaves, we first genome-widely identified the circRNAs and their expression pattern in lifespan of leaves combined with the real-time PCR validation. We then investigated the potential function of circRNAs through miRNA ‘sponge’ and functional prediction of correlated mRNAs.

## Materials and Methods

### Data Source and Identification of Circular RNAs

*Arabidopsis thaliana* genome (TAIR10) were downloaded from the database of Ensembl Plants. A set of publicly available total RNA-seq data of Arabidopsis leaves (GEO accession GSE43616; read length 101 bp) were used for identifying the circRNAs. The dataset includes 14 time points from 4 days after emergence to 30 days at 2-day intervals during the lifespan of leaves. We used MapSplice 2.0 (Wang et al., 2010) to identify circRNAs. The circRNA sequences were extracted according to the method used in CircNet database (Liu Y. C. et al., 2016).

### Identification of Differentially Expressed circRNAs

Those circRNAs that were identified in at least two samples were used for subsequent analyses. The expression levels of circRNAs were determined by the number of identified reads adding one normalized by the total reads in each RNA-seq data set. Log_2_(fold-changes) were calculated using the expression value at other time points divided by the expression value at 4 days for G- to -M stage (the growth -to -maturation stage, 4–18 days)) and 16 days for M- to -S stage (the maturation -to -senescence stage, 16–30 days). Differentially expressed circRNAs were defined as absolute log_2_ (fold-changes) ≥2 based on the method used in Woo et al. (2016).

### Prediction of miRNA Target

We used the following three target prediction methods with default parameters to predict the miRNA targets: (i) psRNA target tool using a score ≤4 for mRNA and a score ≤4.5 for circRNA (Dai and Zhao, 2011); (ii) TAPIR method (Bonnet et al., 2010) used a score ≤5 and ratio of the free energy of the duplex to the free energy of the same duplex having only perfect matches ≥0.7 for mRNA and a score ≤6 and ratio of the free energy of the duplex to the free energy of the same duplex having only perfect matches ≥0.6 for circRNA; (iii) AtmiRNET (Chien et al., 2015) using a score ≥180 for mRNA and a score ≥140 for circRNA. The targets of miRNAs were selected based on age dependent expression changes and having anti-correlation with miRNA (Spearman’s correlation coefficient ≤–0.5).

### Construction of circRNA–miRNA–mRNA Network

We used the miRNAs, mRNAs, and circRNAs separately with 2-, 4-, and 4-fold changes in expression at G- to -M and M- to -S stage to construct the circRNA–miRNA–mRNA pair. The potential connections between circRNA, miRNA, and mRNA were drawn by Cytoscape (version 3.4.0).

### Function Annotation

The function of predicted target mRNAs for circRNAs were analyzed with gene ontology (GO) and KEGG pathways annotation using DAVID (version 6.8).

### Validation of Circular RNAs

Quantitative real-Time PCR (qRT-PCR) was used to validate the expression of circRNAs identified by RNA-seq. The fourth rosette leaves of wild-type Columbia plants grown under long-day conditions (16 h light: 8 h dark) were harvested at 4 h after light-on at 4, 16, and 28 days. Total RNAs were extracted from leaves using Total RNA Purification Kit (GeneMark). 0.2 μg of total RNA was reverse transcribed into cDNA with random primer. The primers were designed according to the exon sequence adjacent to the circulation junction. The primers sequences are listed in Supplementary Table 4. The cDNA sample (1.8 μl), primer pairs (5 μM, 1 μl), SYBR qPCR Master Mix from Vazyme (10 μl) and RNAase-free water (7.2 μl) in combination were used in real-time PCR reactions. The annealing temperature of PCR cycles was 55°C in all reactions and for different primer pairs. Gene expression was normalized to that of actin. The real-time PCR assays were performed in three biological replicates and the results were presented as mean + SD.

## Results

### Overview the circRNA in Arabidopsis Leaves

We used MapSplice (version 2.0) to identify the circRNAs. A set of publicly available RNA-seq data of total RNAs from Arabidopsis leaves were obtained from GSE43616 for identifying of circRNAs. We identified a total of 168 circRNAs (Supplementary Table 1). We further compared our identified circRNAs with the ones of *Arabidopsis thaliana* in PlantcircBase (Chu et al., 2017) database, and found 40 novel circRNAs.

Among those identified circRNAs, 158 (94.1%) of them were generated from the exons of corresponding genes. Interestingly, splice sites of 134 (84.8%) circRNAs of these 158 exonic circRNAs were perfectly located in both ends of exons. In addition, both splice sites of 8 (5.1%) circRNAs were located in internal site of exons, one site of 16 (10.1%) circRNAs located in the end of exons, the other site located in the internal site of exons. 142 (89.9%) exonic circRNAs included 1–5 host-gene-derived exons. We did not identify any circRNAs generated from introns. We found 10 (5.9%) circRNAs located in the intergenic regions (**Table 1**). Among all the identified circRNAs, we found three circRNAs generated from Chloroplast genome and they all located in the intergenic region. However, we did not detect the circRNAs generated from Mitochondria genome. These results indicate that circRNAs of Arabidopsis can be generated from diverse genomic regions through different splicing modes. We also found that some genes may produce more than one circRNAs through alternative back splicing or the combination of different exons.

**Table 1:** Circular RNAs in *Arabidopsis thaliana* leaves.

Type of CircRNA	Number	Percentage
Both splice sites exactly matched to boundaries of exons	134	79.8
Both splice sites located in internal region of exons	8	4.8
One site exactly matched to exon boundary, and the other site located in the internal region of one exon	16	9.5
Intergenic	10	5.9
Total	168	100

**FIGURE 1:**
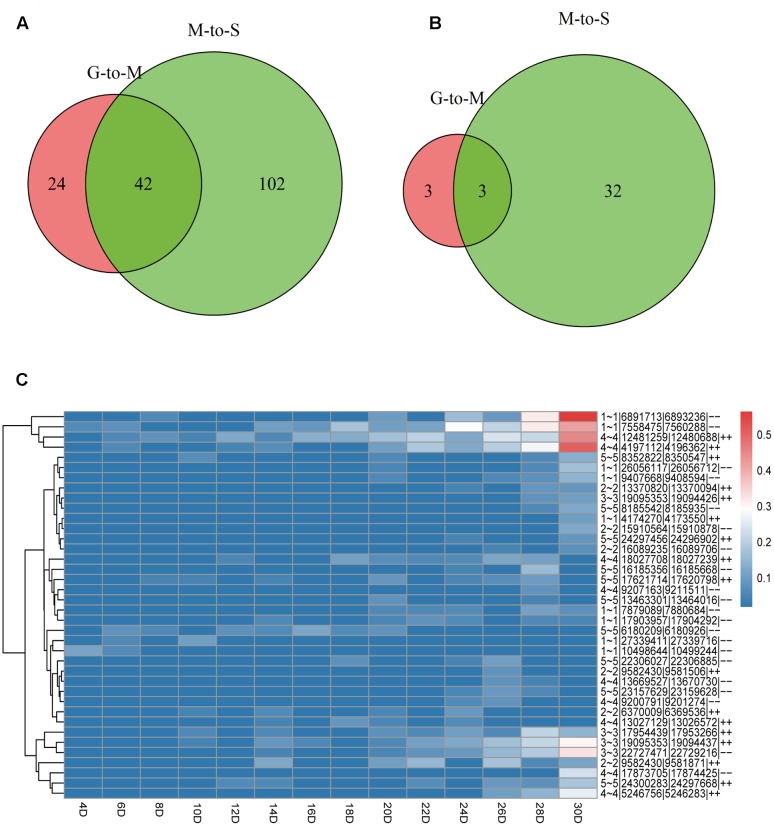
The distribution of circRNAs during the lifespan of leaves and expression pattern of DE-circRNAs. **(A)** Numbers of circRNAs at G- to -M and M- to -S. **(B)** Numbers of DE-circRNAs at G- to -M and M- to -S. **(C)** Heat maps showing the expression patterns of 38 DE-circRNAs during leaf growth, maturation and senescence.

Based on the number of back-spliced reads identified, we found that only 7 (4.2%) circRNAs were detected in more than 10 samples. 104 (61.9%) circRNAs were only detected in one sample. We separately identified a total of 66 (39.3%) and 144 (85.7%) circRNAs during G- to -M and M- to -S stages, including 42 circRNAs in both stages, suggesting that more circRNAs uniquely expressed in the stage of leaves’ senescence (**Figure 1A**). The result suggests that a large portion of circRNAs only expressed at a specific time point.

We examined the function of host genes that encode circRNAs. GO enrichment analysis for the host genes showed that they were mainly involved in the following biological processes, such as embryo development, negative regulation of cellular metabolic process, reproductive system development and tetrapyrrole metabolic process. Thus circRNAs could be generated from diverse genes with different functions.

### Identification of Differentially Expressed circRNAs

Only those circRNAs that expressed in at least two samples were considered and 64 circRNAs were kept for subsequent analyses (Supplementary Table 2). We then conducted differential expression calling based on the expression of circRNAs during growth, maturation and senescence stages. With the threshold of 4 fold changes, 6 circRNAs were differentially expressed at G- to -M stage (5 of them were up-regulated and one was down-regulated), while 35 circRNAs were differentially expressed at M- to -S stage (34 of them were up-regulated and one was down-regulated) (**Figures 1B,C** and Supplementary Table 3). And 3 circRNAs were differentially expressed in both G- to -M and M- to -S stages. More circRNAs showed differential expression at M- to -S stage compared to G- to -M stage and almost all of them were up-regulated. Thus circRNAs showed an upregulation trend with senescence, suggesting that circRNAs may play important roles during the senescence stage of leaves.

### Validation of Circular RNAs

**FIGURE 2:**
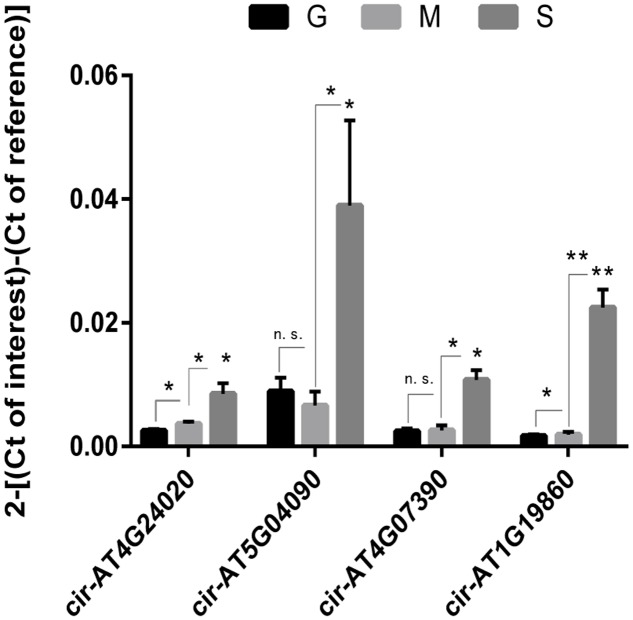
Validation of the expression pattern of four highly expressed circRNAs by real-time PCR. Real-time PCR was used to verify the expression of four highly expressed circRNAs during leaf growth, maturation and senescence. Three replicates were made. The expression of actin gene was used as the internal reference. Asterisk on S bars reflect significant changes versus G (*n* = 3). Error bars represent standard error of the mean. n.s., not significant. ^∗^*P* < 0.05; ^∗∗^*P* < 0.01.

To further validate circRNAs, we carried out real-time PCRs to verify the expression patterns of 4 highly expressed circRNAs which contain one novel circRNA Cir-AT1G19860. The primers were designed according to the exonic sequence adjacent to the circulation junction (Supplementary Table 4). Real-time PCRs showed consistent expression patterns with the RNA-seq results. As shown in **Figure 2**, with the development of the leaves, four circRNAs were up-regulated in expression and the senescence stage is the highest.

### The Expression of Up-Regulated circRNA during Senescence Are Independent of Host Gene Expression to a Certain Degree

Since most circRNAs are generated from the protein-coding genes, we further examined that whether abundance changes between the circRNA and transcript of the host gene are correlated. We found that no up-regulated circRNAs at G- to -M stage had corresponding up-regulated linear RNAs and only 9/34 up-regulated circRNAs at M- to -S stage also had their linear RNA expression concomitantly increased. Previous study also showed that the circRNA abundance changes were independent of the general transcription from their host genes to a certain degree (Gruner et al., 2016). Our result suggests that upregulation of circRNAs during leaves senescence stage are not the results of the up-regulated transcript of corresponding host genes and the circRNAs may play important roles in the senescence of Arabidopsis leaves.

### Construction of circRNA–miRNA–mRNA Network

**FIGURE 3:**
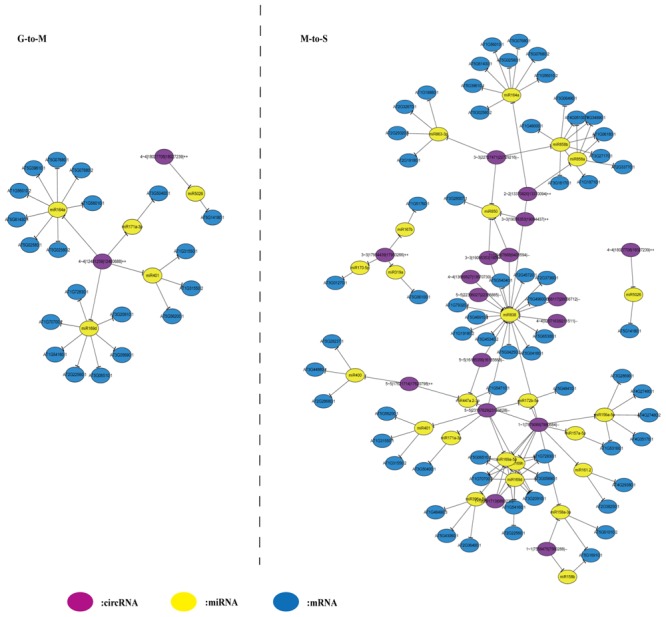
The circRNA-microRNA-mRNA network at G- to -M and M- to -S. The circRNA-microRNA-mRNA network at G- to -M consists of 20 pairs. The circRNA-microRNA-mRNA network at M- to -S consists of 220 pairs.

To construct the circRNA–miRNA–mRNA network, we first downloaded the differentially expressed transcripts and miRNA used by Woo et al. (2016) for analysis. The targets of 52 differentially expressed miRNAs of differentially expressed transcripts and circRNAs were predicted using three different tools. We identified circRNA–miRNA–mRNA pairs that miRNA showed anti-correlation (spearman correlation ≤ –0.5) with corresponding targets (circRNAs and mRNAs) in expression (20 and 220 circRNA–miRNA–mRNA pairs at G- to -M and M- to -S, respectively) (Supplementary Table 5). Then the potential connections between circRNAs, miRNAs and mRNAs were explored by Cytoscape. As shown in **Figure 3**, the regulatory network during M- to -S stage is more complex compared with G- to -M stage, suggesting that circRNAs are active in expression regulation during the senescence process.

### Function Annotation

**FIGURE 4:**
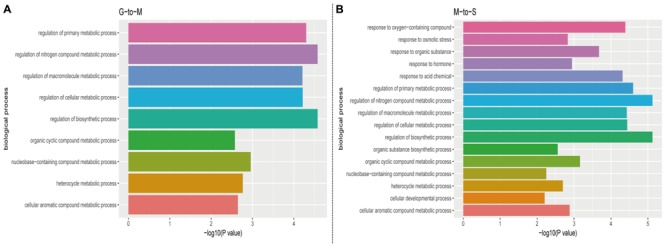
The biological process profile of the target genes of the differentially expressed circRNAs during **(A)** G- to -M stage **(B)** M- to -S stage. (*P*-value < 0.01).

The GO analysis was performed by DAVID (version 6.8) to explore the function of predicted mRNA of circRNA. The result showed that the biological processes at G- to -M stage belong to a variety of metabolic processes, such as regulation of biosynthetic process (GO: 0009889) and regulation of cellular metabolic process (GO: 0031323). In addition to including a variety of metabolic processes, we also found response to hormone (GO: 0009725), response to osmotic stress (GO: 0006970), response to organic substance (GO: 0010033) and other biological process at M- to -S stage (**Figure 4**). The GO analysis on predicted mRNAs showed that the targets of differentially expressed circRNAs during leaves development and senescence were associated with various biological processes and circRNAs may contribute distinctly in these two stages by involving in different biological processes.

**FIGURE 5:**
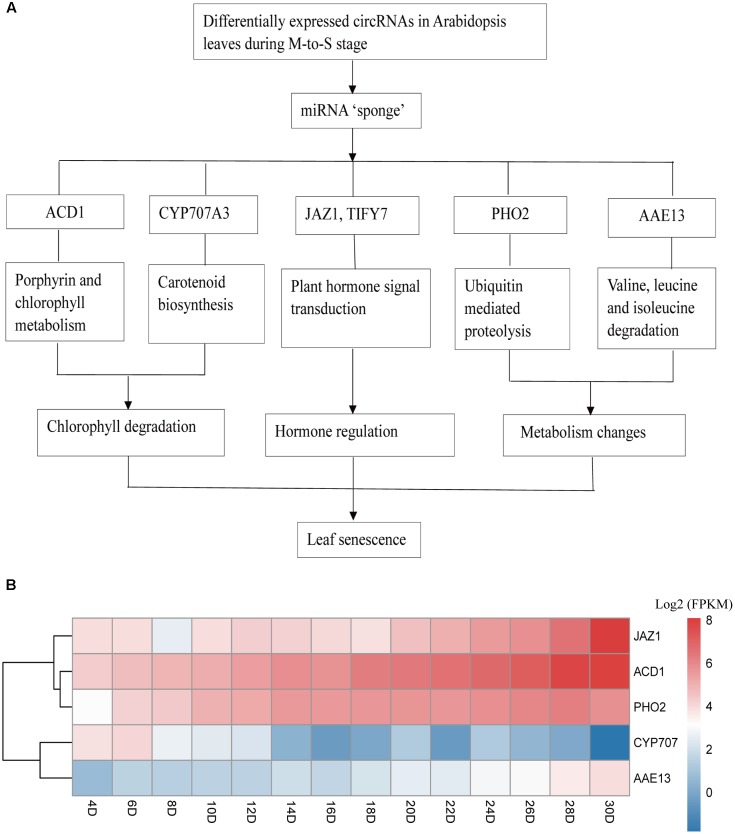
Possible regulatory mechanism involving differentially expressed circRNAs and their target genes in Arabidopsis leaves at M- to -S stage. **(A)** ACD1, Pheophorbide a oxygenase family protein with Rieske 2Fe-2S domain-containing protein; CYP707A3, cytochrome P450, family 707, subfamily A, polypeptide; JAZ1, jasmonate-zim-domain protein 1; TIFY7, TIFY domain/Divergent CCT motif family protein; PHO2, phosphate 2; AAE13, AMP-dependent synthetase and ligase family protein. **(B)** The expression profile of five target genes during the lifespan of Arabidopsis leaves.

The KEGG pathway analysis was also conducted to further explore the function of predicted mRNA of circRNA and 11 pathways were obtained. Among the obtained KEGG pathways, some of them are related with the leaves development and senescence in Arabidopsis or other plants. The result showed there were only one pathway, plant hormone signal transduction at G- to -M stage. Compared with G- to -M stage, the situation was more complicated at M- to -S stage. For example, as shown in **Figure 5**, the Porphyrin and chlorophyl metabolism related genes included Pheophorbide a oxygenase family protein with Rieske 2Fe-2S domain-containing protein (ACD1). Carotenoid biosynthesis related genes included cytochrome P450, family 707, subfamily A, polypeptide 3(CYP707A3). Two predicted target mRNAs were involved in the plant hormone signal transduction pathway, including TIFY domain/divergent CCT motif family protein(TIFY7), and jasmonate-zim-domain protein 1 (JAZ1). Besides, the predicted target mRNAs of differentially expressed circRNAs also included ubiquitin mediated proteolysis related phosphate 2 (PHO2) and Valine, leucine, and isoleucine degradation related AMP-dependent synthetase and ligase family protein (AAE13). In summary, they belong to three major parts, chlorophyll degradation, hormone transduction and metabolic changes at M- to -S stage.

## Discussion

Although the circRNAs have been widely studied in human (Chen et al., 2016; Peng et al., 2016), systematic studies of plant circRNAs are just beginning. The development and senescence of leaves are very complex processes, which involve thousands of genes and multiple layers of regulation. Our results demonstrate that circRNAs display an upregulation trend in the lifespan of Arabidopsis leaves, and may exercise important function as new post-transcriptional regulators during leaves development and senescence process.

We found that the circRNAs can be generated from diverse genomic regions through different splice modes and 94.1% of them were from exons, which is consistent with previous study (Ye et al., 2015; Meng et al., 2016). Our results first reveal that circRNAs showed the developmental-specific expression pattern in Arabidopsis leaves. Specifically, some circRNAs only expressed in a particular stage, for example, circ-AT1G29965 was only expressed in the early stage of leaf growth, while circ-AT5G18590 was only detected in the mature stage of leaf growth, and circ-AT4G08300 was expressed only in the senescence process. CircRNAs specifically expressed at a specific developmental stage could have their roles at that certain developmental stage. The results indicate that the circRNAs could be the important functional regulator in the lifespan of Arabidopsis leaves.

Recent study showed a genome-wide trend for increased circRNA expression in aging brain of mice (Gruner et al., 2016). Interestingly, differential expression analysis revealed that circRNAs were also mainly up-regulated in Arabidopsis leaves and the circRNAs display an upregulation trend during the leaves senescence, indicating that circRNA may play important roles in the senescence process. Although the expressions of most of the circRNAs are certainly independent of their host gene expression during senescence of leaves, 9 up-regulated circRNAs at M- to -S stage had their linear RNA expression concomitantly increased. Exon-intron circRNAs could interact with U1 snRNP and promote transcription of their parental genes in human cells (Li et al., 2015). In *Oryza sativa*, circRNAs can upregulate the expression of their parent genes (Wang et al., 2017). Therefore, this might be the reason that resulted in concomitantly increased expression between those 9 circRNAs and their linear RNAs.

The 35 differentially expressed circRNAs (DE-circRNAs) during M- to -S stage were larger than that of G- to -M stage (6 DE-circRNAs), suggesting that circRNA may play important roles during the senescence of leaves. Some environmental factors could influence the leaf senescence, such as, oxidative stress, drought and nutrient deficiency. Previous studies have shown the changes of circRNAs under stress. For example, 62 circRNAs are differentially expressed under dehydration stress and target drought-specific miRNAs are associated with photosynthesis and hormone signal pathway in wheat (Wang et al., 2016). Moreover, a number of circRNAs in rice and barley have also been reported to respond to nutrients stress such as phosphate, iron, and, zinc (Ye et al., 2015; Darbani et al., 2016). Therefore, plant circRNAs could play a regulatory role at the senescence stage of leaves in view of the relationship between senescence and stress.

Since circRNAs can function as the sponge of miRNAs, we used the predicted mRNAs that shared the same targeting miRNAs with corresponding DE-circRNAs to explore the function of circRNAs during leaf development and senescence. Compared to G- to -M stage, we found some other biological processes were observed at M- to -S stage, such as response to hormone (GO: 0009725), response to osmotic stress (GO: 0006970) and response to organic substance (GO: 0010033). These biological processes are associated with senescence process to a certain extent (Woo et al., 2013), further implying that the circRNAs could have a potential role in the senescence phase.

We found that some predicted target genes of circRNAs could play important roles during leaf senescence. For example, ACD1 (accelerated cell death) is related with Porphyrin and chlorophyll metabolism, while JAZ1 is associated with Plant hormone signal transduction. ACD1 can encode a pheide a oxygenase (PAO) to contribute to the breakdown of chlorophylls during the senescence of the leaves (Pruzinska et al., 2005). JAZ1 is a nuclear-localized protein involved in jasmonate signaling and jasmonic acid (JA) is the endogenous regulatory substances that have the roles of inhibition of plant growth and promoting senescence in plants including Arabidopsis (He et al., 2002). Our study proposed that the circRNAs might play a role during senescence stage in Arabidopsis leaves by mediating the Porphyrin and chlorophyll metabolism and hormone signal pathway. In addition, the circRNAs might contribute to the degradation of the nutrients produced in the growth phase of the leaf and their redistribution to developing seeds or other parts of plants.

In summary, we identified 168 circRNAs in Arabidopsis leaves including 40 novel ones, and revealed that circRNAs may tend to accumulate in senescence process. Moreover, the expression of circRNAs during senescence is independent of host gene expression to a certain degree. Our analyses of circRNA–miRNA–mRNA network indicate that the circRNAs were involved in senescence-responsive processes, such as porphyrin and chlorophyll metabolism and plant hormone signal transduction. Our study reveals the circRNAs may function as new post-transcriptional regulators in the senescence stage of *A. thaliana* leaves.

## Author Contributions

TL conceived and designed the study. TL performed the experiments, analyzed the data, and wrote the manuscript. LZ and GC assisted with discussing of the results. GC and TS helped to revise the manuscript. All authors approved the final manuscript.

## Conflict of Interest Statement

The authors declare that the research was conducted in the absence of any commercial or financial relationships that could be construed as a potential conflict of interest.
